# Influence of Testosterone Metabolites on Song-Control System Neuroplasticity during Photostimulation in Adult European Starlings (*Sturnus vulgaris*)

**DOI:** 10.1371/journal.pone.0040060

**Published:** 2012-07-06

**Authors:** Zachary J. Hall, Scott A. MacDougall-Shackleton

**Affiliations:** 1 Department of Biology, Advanced Facility for Avian Research, University of Western Ontario, London, Ontario, Canada; 2 Department of Psychology, Advanced Facility for Avian Research, University of Western Ontario, London, Ontario, Canada; University of Lethbridge, Canada

## Abstract

The song-control system is a network of discrete nuclei in the songbird brain that controls the production and learning of birdsong and exhibits some of the best-studied neuroplasticity found in the adult brain. Photoperiodic growth of the song-control system during the breeding season is driven, at least in part, by the gonadal steroid testosterone. When acting on neural tissue, however, testosterone can be metabolized into 5α-dihydrotestosterone (DHT) or 17β-estradiol (E2), which activate different hormonal signaling pathways. By treating adult starlings with both testosterone metabolites and metabolite antagonists, we attempted to isolate the effects of androgen and estrogen treatment on neuroplasticity during photostimulation in male and female European starlings (*Sturnus vulgaris*). Photostimulation resulted in a large HVC volume typical of the breeding season in all treatments independent of hormone treatment. E2 had additional effects on HVC growth by reducing neuron density and enhancing early survival of new neurons recruited to HVC in females but did not significantly affect HVC volume. Conversely, DHT reduced the migration of new neurons, assessed by the expression of doublecortin, to HVC. DHT also increased syrinx mass and maintained RA (robust nucleus of the arcopallium) cytoarchitecture in the presence of aromatase inhibitors. In addition, we document the first evidence of sex-specific neuroplastic responses of the song-control system to androgens and estrogens. These findings suggest that the contributions of DHT and E2 signaling in songbird neuroplasticity may be regulated by photoperiod and that future studies should account for species and sex differences in the brain.

## Introduction

Some of the most extreme adult neuroplasticity in vertebrates occurs in the song-control system of songbirds, a circuit of discrete nuclei underlying birdsong production and learning. In most temperate-zone songbirds studied, this plasticity is entrained to seasonal changes in photoperiod throughout the year. Specifically, vernal increases in photoperiod during the breeding season induce the volumetric growth of song-control system nuclei including HVC (used as a proper name), the robust nucleus of the arcopallium (RA), and Area X. Conversely, photorefractoriness at the end of breeding is associated with regression of song nuclei [1]. Cytoarchitectonically, changes in neuron size, neuron density, synaptogenesis, angiogenesis, neuron turnover and incorporation, and glia density produce larger song-control system nuclei volumes in the breeding season [2]–[4]. Furthermore, these anatomical changes parallel seasonal changes in song [5] and electrophysiology [6]. The magnitude and behavioral correlates of neuroplasticity in the song-control system make songbirds an ideal model for studying the physiological control of adult neuroplasticity.

The predominant physiological cue mediating photoperiodic song-control system growth is thought to be the gonadal steroid testosterone (T). Vernal increases in photoperiod correlate with both an increase in plasma T and growth of the song-control system [5]. Conversely, reduction of plasma T via castration induces regression of song-control system nuclei that is reversed with subsequent T replacement [7], [8] and exposure to long days characteristic of the breeding season (photostimulation) in castrated adult male white-crowned sparrows does not stimulate song-control system growth [9]. Although overwhelming evidence has implicated T’s important role in controlling song-control system growth, other factors have been found to modulate the efficacy of T to induce such plasticity. In starlings, photoperiodic state significantly regulates T-induced song-control system growth: whereas birds in a prebreeding condition that are sensitive to long days (photosensitive), maximize HVC volume during T treatment, birds in a post-breeding condition in which they are insensitive to long days (photorefractory), treated with T exhibited no significant HVC growth [7]. In the wild, photostimulation by long days is associated with an increased level of plasma T, making it difficult to dissect the influences of these two factors on song nuclei growth. Here, we investigated the contributions of T signaling pathways to song-control system growth during photostimulation.

T can act either directly on target tissue via androgen receptors or indirectly through metabolism into other steroids including 5-dihydrotestosterone (DHT), an androgen more potent than T, and 17β-estradiol (E2), an estrogen metabolized from T by aromatase [10]. Once produced, E2 and DHT are not converted into one another or T [11] and specifically bind to estrogen receptors and androgen receptors, respectively. Both the conversion enzymes [12] and receptors [13] necessary for DHT and E2 metabolism and action are localized in the song-control system. In starlings, androgen receptor mRNA is expressed in HVC, the lateral magnocellular nucleus of the neostriatum, and RA. Estrogen receptor alpha mRNA is expressed in medial HVC and the caudal border of the neostriatum [14]. Thus, the song-system is a target for both estrogens and androgens.

Studies experimentally manipulating DHT and E2 have reported both synergistic [15], [16] and redundant [9] effects on song-control system growth. When administered alone, E2 is known to have neuroprotective properties, reducing neuron turnover in HVC [17], [18]. Additionally, both DHT and E2 administration increased the volumes of HVC, RA, and Area X [9]. However, these studies often assumed that gonadectomy eliminated androgens and estrogens in control birds. It is now known that the brain is capable of producing significant levels of androgens and estrogens [18]–[20] that may modulate exogenous hormone treatments. Additionally, these studies commonly measure only anatomical volume, limiting our understanding of changes in cytoarchitecture and neurogenesis accompanying song-control system plasticity.

In this study, we investigated the contributions of isolated and combined DHT and E2 signaling on song-control system growth during photostimulation, a time when HVC exhibits substantial growth in volume [1]. We isolated DHT and E2 signaling in both male and female adult European starlings (*Sturnus vulgaris*) by simultaneously administering T metabolites and metabolite antagonists and measured volumetric, cytoarchitectonic, and neurogenesis parameters in HVC, Area X, and RA.

## Materials and Methods

### Ethics Statement

All procedures complied with guidelines set out by the Canadian Council on Animal Care (CCAC), and and followed Guidelines to the Use of Wild Birds in Research [21]. This study and all of its protocols were approved under protocol #2009-068 by the Animal Use Subcommittee of the University of Western Ontario.

### Birds

Seventy adult European starlings (*Sturnus vulgaris*; 38 males and 32 females) were trapped near London, Ontario in January 2010, a time when birds are non-reproductive but photosensitive [22]. Birds were group-housed in large indoor aviaries on a short-day light:dark cycle of 7.5∶16.5 h with access to *ad libitum* food for a minimum of one week.

### Surgical Manipulation

Birds received surgery in cohorts comprised of a bird from each treatment group. Each day of surgery, 4 females and 5 males were opportunistically caught from the aviaries and randomly assigned to treatment groups (Table 1; groups: DHT+ E2−, DHT− E2+, DHT− E2−, DHT+ E2+, and control; + refers to hormone administration and – refers to hormone antagonism). Before surgery we recorded bird mass and the proportion of beak yellow (an androgen-dependent trait). All birds were then gonadectomized under general anesthesia (isoflurane) using aspiration and arch-tipped forceps. Before gonadectomy, we visually inspected all gonads to assess the birds’ reproductive condition. All birds had regressed gonads, confirming their non-breeding photosensitive state prior to hormone and photoperiodic treatment. Fadrozole (FAD; Novartis Pharma AG), an aromatase inhibitor used to block E2 synthesis, was administered via osmotic mini-pump calibrated using previous studies (Alzet model 1004; 12.12 mg/kg bird/day; [18]) and inserted into the peritoneal cavity through the gonadectomy incision. Birds not treated with FAD were implanted with mini-pumps filled with saline. DHT (Steraloids), E2 (Sigma), and Flutamide (FLUT, an androgen receptor blocker; Sigma) were administered via Silastic (Dow Corning) implants of sizes adapted from previous studies implanted subcutaneously on the upper back. Specifically, Silastic implants administering DHT were shortened from prior studies to compensate for reports of supra-physiological DHT administration [9]. Conversely, Silastic implants administering E2 were lengthened from prior studies to compensate for reports of low E2 administration ([9]; Table 2). Empty implants were used on control and DHT+ E2− birds to ensure all birds had a minimum of 2 Silastic implants. Mini-pumps and Silastic implants were incubated in saline for 48 h or 24 h, respectively, prior to implantation to ensure consistent administration rates. An injection of bromodeoxyuridine (BrdU; i.p.; 100 mg/kg) was administered to birds during surgery. Following surgery, birds were moved to individual cages maintained on a long-day photoperiod (16∶8 cycle) to induce photostimulation [22].

**Table 1:** Summary of the sample sizes and hormone treatments for each treatment group.

Treatment Group	N	Silastic implants	Osmotic mini-pump
Control	6 M	2 empty	Saline
DHT+ E2+	8 M; 7 F	1 DHT; 2 E2	Saline
DHT+ E2−	8 M; 8 F	1 DHT; 1 empty	FAD
DHT− E2+	8 M; 8 F	2 FLUT; 2 E2	Saline
DHT− E2−	8 M; 8 F	2 FLUT	FAD

Naming for the treatment groups uses + to refer to hormone agonism while – refers to hormone antagonism. M = male; F = female; DHT, 5α-dihydrotestosterone; E2, 17β-estradiol; FLUT, flutamide; FAD, fadrozole.

**Table 2:** Silastic implant dimensions used in surgery and studies from which sizing was referenced and adjusted.

Implant Contents	Length (mm)	Inner diameter (mm)	Outer diameter (mm)	Prior use
Blank	6	1.5	2.0	−
DHT	6	1.5	2.0	Adapted from [9]
E2	12	1.5	2.0	Adapted from [9], [18]
FLUT	14	1.5	2.0	[55]

DHT, 5α-dihydrotestosterone; E2, 17β-estradiol; FLUT, flutamide.

On day 1 post-surgery, birds were administered an additional injection of BrdU (i.p., 100 mg/kg). Birds were inspected visually on a daily basis to check the presence of implants. Missing implants were replaced within the same day and the incision was re-sealed (n = 2 birds). One female bird (DHT+ E2+) died during treatment and was not replaced (n = 7 in this treatment group). On day 10 post-surgery, beak color was visually inspected and graded along a 5 point scale (0%, 25%, 50%, 75%, or 100% from fully black to fully yellow) and a 450 µl blood sample was collected from the brachial vein of each bird for later confirmation of hormone administration.

### Hormone Assays

Both DHT and E2 levels were measured in plasma using DHT and E2 enzyme-immunoassays (DHT: IBL IB59116; Phoenix Airmid Biomedical; E2: Salimetrics 1–3702; Salimetrics). The lowest point on the DHT standard curve was 0.1 pg DHT per well. Intra-assay variation was 3.68%, and interassay variation was 3.21% (high control only; n = 2 assays). The lowest point on the E2 standard curve was 0.1 pg E2 per well. Intra- assay variation was 6.18%, and interassay variation was 7.59% (low control) and 7.69% (high control; n = 2 assays). For both assays standard curves were linear through the lowest standard on the curve, but to be conservative we calculated sensitivity as equivalent to the lowest standard on the curve (E2∶1 pg/mL, DHT: 2 pg/mL).

### Tissue Collection and Processing

On day 21 post-surgery, body mass and beak color were recorded. Birds were deeply anesthetized with isoflurane and transcardially perfused with phosphate buffered saline (PBS; pH 7.4) followed by 4% buffered paraformaldehyde. The brain was dissected from the skull, submersed in 4% paraformaldehyde overnight, and cryoprotected in 30% sucrose in PBS for 48 h before being frozen on pulverized dry ice and stored at −80°C. Birds were dissected to confirm complete gonadectomy and the presence of all implants. One male and one female were found to have only a partial gonadectomy. Both birds were statistically confirmed to not be outliers in their respective treatment groups before inclusion for intergroup statistical analysis. Previous reports caution that a survival period of 15–22 days post treatment may cloud quantification of neuronal recruitment, as a significant wave of cell death following recruitment occurs in this period in adult male canaries [23]. However, Absil et al. [24] found that a survival time of 22 days was suitable to quantify HVC neuronal recruitment in adult European starlings. Therefore, we expected no difficulties using similar quantification following 21 days of treatment.

Brains were sectioned (40 µm) in the coronal plane using a cryostat and sections collected in 4 alternating series. In each series, every fourth section of HVC, Area X, and RA and every twelfth section of the telencephalon were collected.

### Antibody Characterization (Table 3)

NeuN is a neuron-specific nuclear protein expressed in most mature neurons types [25]. We labeled NeuN with the Millipore MAB377 antibody that has been previously validated to label the nucleus and cytoplasm of mature neurons in the songbird song-control system [26]. Our immunohistochemical labeling of NeuN+ neurons in HVC, RA, and Area X labeled mature neuron soma with absent neurite staining and appeared identical to previous reports of NeuN staining in the song-control system [26]. As a control, spare sections from 5 randomly selected starlings were processed for NeuN staining with the primary antibody omitted: staining was absent in this tissue.

**Table 3:** Characterization of antibodies used to label neuroanatomy in adult European starling brains.

Antibody Name	Immunogen	Manufacturer and Cat. No.	Species raised in	Monoclonal/Polyclonal
Neuron-specific NuclearProtein (NeuN)	Purified cell nucleifrom mouse brainneurons	Millipore MAB377	Mouse	Monoclonal
Doublecortin (DCX)	Peptide mapping atC-terminus (C-18)of Doublecortin ofhuman origin(genetic mapping to Xq23)	Santa Cruz Biotechnologysc-8066	Goat	Polyclonal

Although birds were injected with BrdU, the preferred primary antibody used in our lab for immunolabeling was discontinued by the supplier. Thus, to assess neuron recruitment tissue was immunostained to visualize expression of doublecortin (DCX), an endogenous microtubule-associated protein expressed exclusively in immature neurons in the songbird telencephalon [27], [28]. Labeling DCX provides the advantage of visualizing immature neurons over the entire treatment period, instead of only a subset of new neurons generated during BrdU injection. Our DCX staining using the Santa Cruz Biotechnology sc-8066 antibody labeled two neuronal phenotypes: the first cell type were fusiform cells identified by their long, bipolar cell body identified as migratory neurons. A second population of spherical, multipolar cells thought to represent recruited neurons that have begun integration into existing neural circuits were also labeled. Both neuronal phenotypes were localized to HVC, the nidopallium surrounding HVC, Area X, the striatum, and arcopallium with no staining in RA as in previous reports of DCX expression in the song-control system [16], [27], [28]. As a negative control, spare sections from 5 randomly selected starlings were processed for DCX staining with the primary antibody omitted: staining was absent in this tissue.

### NeuN Immunohistochemistry

To stain for NeuN, tissue was washed twice in PBS before being incubated in 0.5% H_2_O_2_ in PBS for 30 minutes at room temperature to eliminate endogenous peroxidase. Following another pair of PBS rinses, tissue was incubated in 10% Normal Goat Serum (Vector Laboratories) in 0.3% Triton X-100 (Sigma) in PBS (0.3% PBS/T) for 60 minutes at room temperature. Tissue was then moved directly from the serum into a solution of NeuN primary mouse antibody (diluted 1∶2000 in 0.3% PBS/T, Millipore MAB377) before being incubated at 4°C for 21 hours. On the next day, tissue was washed twice in 0.1% PBS/T and incubated with biotinylated goat anti-mouse secondary antibody (diluted 1∶250 in 0.3% PBS/T; Vector Laboratories) for 1 hour at room temperature. After two rinses in 0.1% PBS/T, tissue was incubated in ABC Elite avidin-biotin horseradish-peroxidase complex (Vector Laboratories) for 1 hour. Following a final pair of rinses in 0.1% PBS/T, tissue was reacted with 0.04% diaminobenzidene solution (Sigma FastDAB) for 90 seconds to visualize antibody-avidin-biotin complexes and then rinsed 4 times with PBS. Tissue sections were then mounted on Superfrost Plus microscope slides (VWR), serially dehydrated, cleared, and cover-slipped with Permount (Fisher).

### Doublecortin Immunohistochemistry

We modified previous immunohistochemical protocols for visualizing DCX [28]. Briefly, tissue for DCX immunostaining was removed from cryoprotectant and washed in PBS, incubated in 0.5% H_2_O_2_ in PBS for 30 minutes, blocked in 10% Normal Horse Serum (Vector Laboratories) in 0.3% PBS/T for 60 minutes and then incubated with DCX primary goat antibody (diluted 1∶250 in 0.3% PBS/T, Santa Cruz Biotechnology sc-8066) overnight at 4°C. On the next day, tissue was washed in 0.1% PBS/T, incubated with biotinylated horse anti-goat secondary antibody (diluted 1∶250 in 0.3% PBS/T; Vector Laboratories) for 1 hour followed by ABC Elite avidin-biotin horseradish-peroxidase complex (Vector Laboratories) for 1 hour. Tissue was then reacted with 0.04% diaminobenzidene solution (Sigma FastDAB) to visualize immunolabelled DCX. Tissue sections were then mounted on Superfrost Plus microscope slides (VWR), serially dehydrated, cleared, and cover-slipped with Permount (Fisher).

### Neuroanatomical Measurements

**Figure 1:**
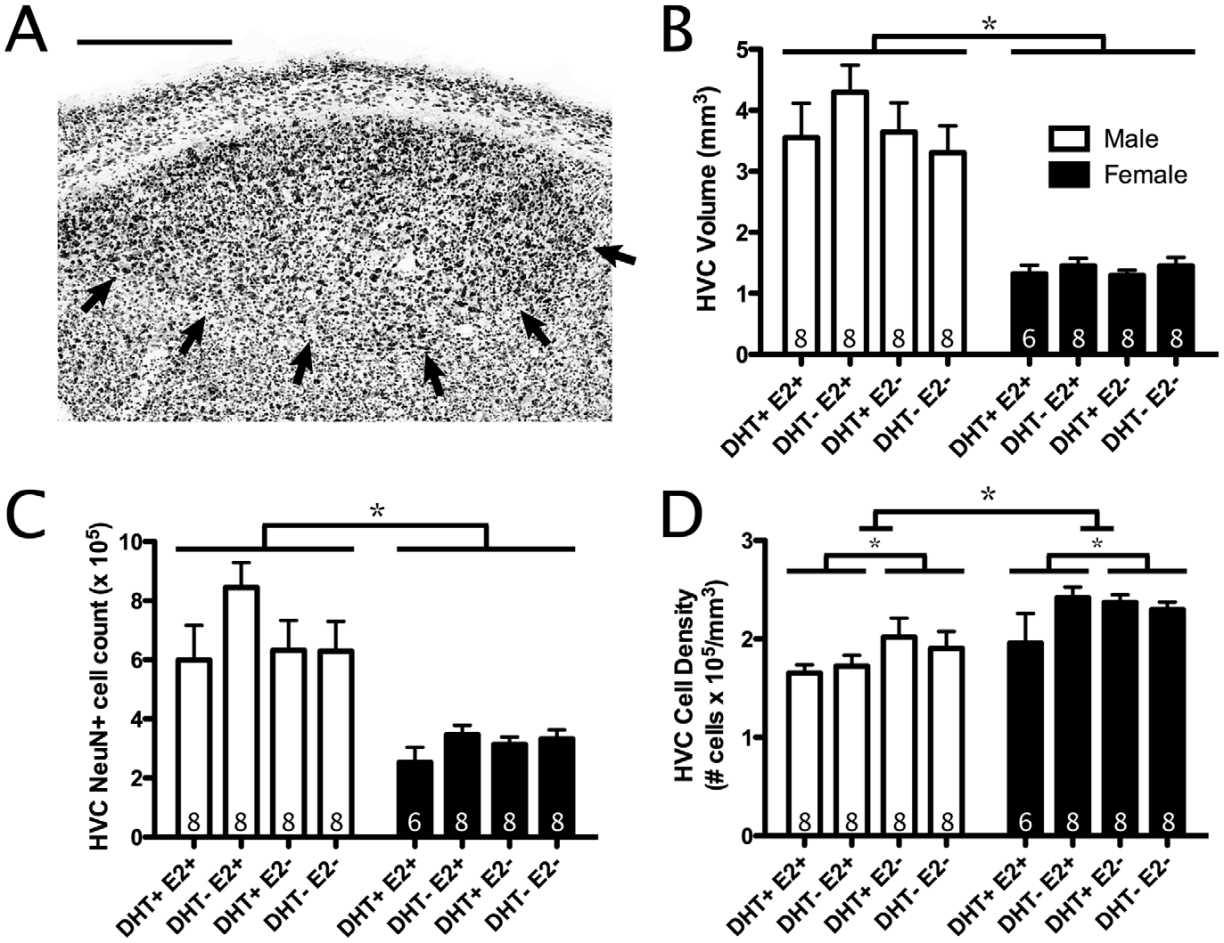
Effects of testosterone metabolites on anatomy of HVC. A) Photomicrograph of HVC in NeuN-stained (NeuN+) tissue. Ventral border indicated by black arrows. Scale bar = 500 µm. B) Volumes of HVC across treatments. C) NeuN+ cell count in HVC. D) Density of NeuN+ cells in HVC. All bars represent mean ± SEM. * <0.05.

**Figure 2:**
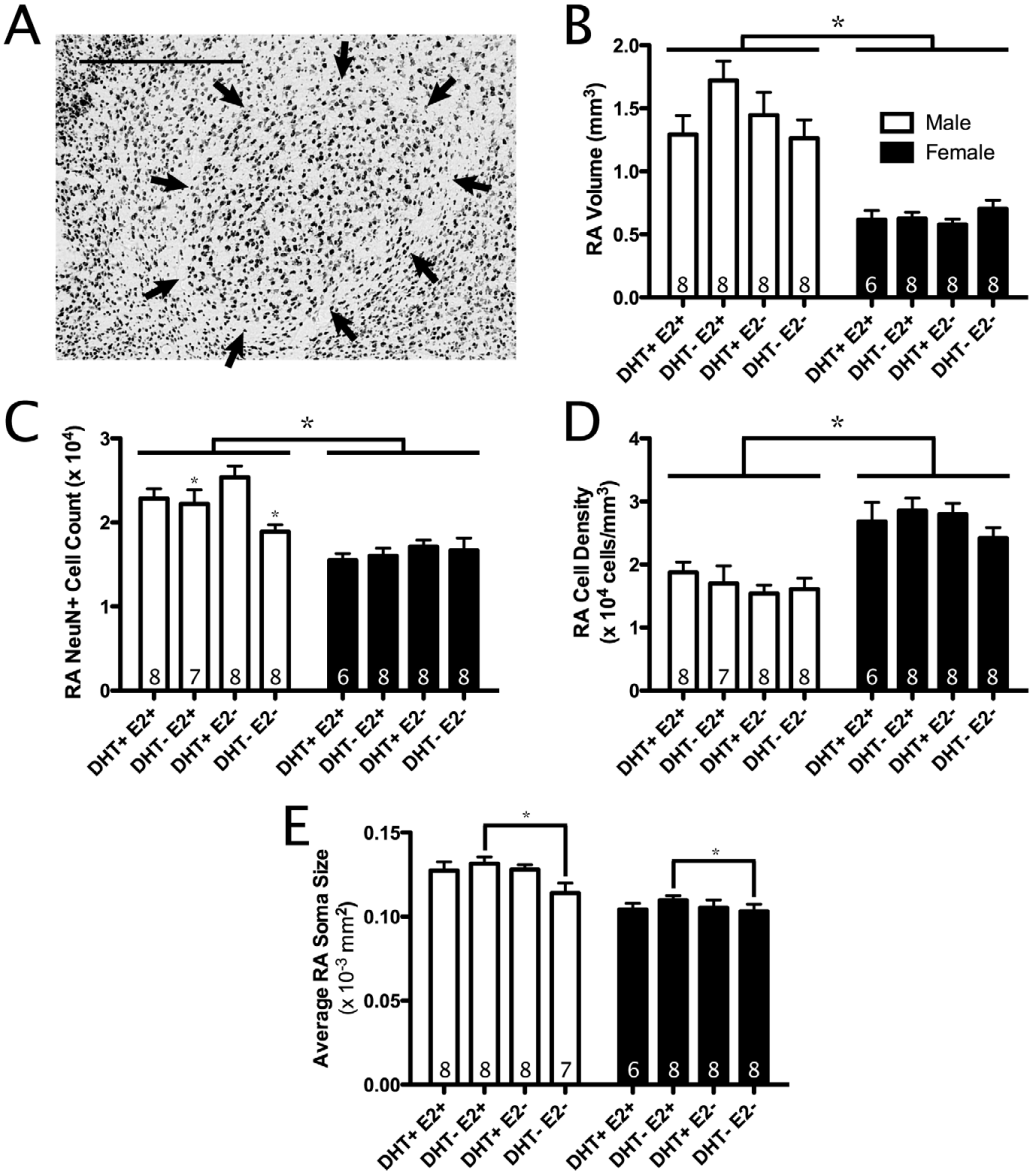
Effects of testosterone metabolites on anatomy of RA. A) Photomicrograph of RA in NeuN-stained (NeuN+) tissue. Nuclear borders indicated by black arrows. Scale bar = 500 µm. B) Volumes of RA across treatments. C) NeuN+ cell count in RA. D) Density of NeuN+ cells in RA. E) Average NeuN+ cell soma size in RA. All bars represent mean ± SEM. * <0.05. RA, robust nucleus of the arcopallium. In C), asterisks without connecting lines mark significant differences found using post-hoc analysis.

In tissue immunostained for NeuN, song nuclei are delineated visually by their larger and darker soma and different cell density than surrounding neural substrate (Fig. 1A, 2A, 3A). For HVC, Area X, and RA, images were captured with a Leica DFC420 C camera mounted on a Leica DM5500 B microscope using a 1.25× objective lens for HVC and Area X and a 5× objective lens for RA. An experimenter blind to the bird’s treatment group traced song nucleus perimeters using Leica Application Suite software to yield cross-sectional areas. Volume was calculated using the formula for a frustrum (truncated cone) accounting for sampling interval (160 µm) and consecutive frustra volumes were summed to estimate total volume. If processing or tissue damage prevented the delineation of a nucleus in a section, area was estimated using the average area of sections before and after the missing section. Telencephalon images were captured using a high-resolution (2400 dpi) flatbed scanner with a transparency adapter. The perimeter of every 14^th^ telencephalon image was traced using NIH ImageJ to calculate cross-sectional areas. As above, frustrum volumes between sections were calculated and summed. If tissue damage or loss of tissue during processing prevented measurement of a telencephalon section, the next closest section was used and sampling interval was adjusted.

**Figure 3:**
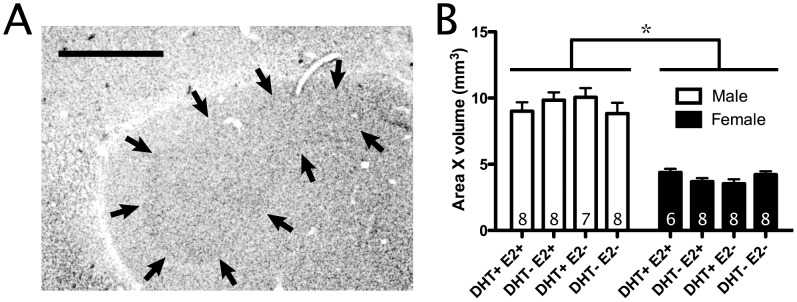
Effects of testosterone metabolites on anatomy of area X. A) Photomicrograph of Area X in NeuN-stained tissue. Nuclear borders indicated by black arrows. Scale bar = 500 µm. B) Volumes of Area X across treatments. All bars represent mean ± SEM. * <0.05.

Average soma size in RA was sampled using NeuN-reacted tissue. Images of RA were captured as above using a 20× objective lens at three consecutive sections (intersection interval = 160 um) in one, randomly selected hemisphere of each bird. Using ImageJ software, images were desaturated and a fixed brightness threshold was applied to highlight immunoreactive soma from background staining. The *analyze particles* function of ImageJ was used to measure cell area of highlighted cells. To exclude particulate matter and overlapping cells, only objects larger than 800 pixels^2^ with a minimum sphericity value of 0.65 were measured. The areas of 30 randomly-selected cells were sampled from each tissue section to yield a total of 90 cells for each bird.

### Stereological Cell Counting

Tissue stained for NeuN expression was also used to estimate the total number of cells in both HVC and RA. We did not sample cell number in Area X because extreme overlap of labeled cells prohibited accurate cell counts using the section thickness selected in this study. It should be noted that a recent study has shown that a thinner section thickness can be used to estimate cell number in this region [29].

Following [26], we adopted the stereological optical fractionator technique to sample the total number of cells in HVC and RA. Analysis began on every 8th section using a 20× objective lens and Leica Application Suite to manually generate either a 320 µm x 320 µm or 160 µm×160 µm sampling grid over HVC or RA, respectively. Because of the small size of RA, the sampling frequency in the x and y plane was increased when sampling RA to ensure a sufficient number of cells was sampled to yield an accurate cell count. Using a 63× oil immersion objective lens (NA = 1.25), the stage was positioned over the first sampling location in the grid. At each sampling location, cells were counted within a 30 µm×30 µm sampling square overlaid on the computer monitor. Cells were only counted when the top of the cell came into focus while focusing through the thickness of the section in 1 µm focal plane steps. Additionally, cells were not counted if their tops fell within the top and bottom 2 µm of the section thickness (guard zones). Because of shrinkage during processing, section thickness ranged from 8 to 16 µm. Using these parameters, 3 to 5 sections of both the right and left HVC and 3 to 6 sections of both the right and left RA were analyzed stereologically. This stereological paradigm sampled between 102 and 855 NeuN cells in HVC and 83 to 213 NeuN cells in RA. The following equation was then used to estimate the total cell count in HVC and RA (with corresponding variable values for HVC and RA summarized in Table 4):
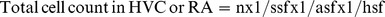



As mentioned above, stereological sampling including cell counts in both hemispheres, yielding a total cell count of cells in both right left song nuclei summed together. Once the total cell count in HVC and RA was calculated for each bird, cell counts were divided by the summed nucleus volume to yield an estimated cell density for each nucleus used in statistical analysis between treatment groups.

**Table 4:** Explanations and numerical values of variables used to stereologically calculate neuronal population sizes.

Variable	Name	Calculation	Value used for HVC	Value used for RA
n	Total number of cells sampled	Counted using stereological sampling	−	−
ssf	Section sampling frequency	1/(frequency of sampled sections)	1/8	1/8
asf	Area sectioning frequency	(Area of sampling square)/(area of the square delineated by adjacent sampling squares)	(30 µm×30 µm)/(320 µm×320 µm)	(30 µm×30 µm)/(160 µm×160 µm)
hsf	Optical dissector height	(average section thickness –4 µm)/(averagesection thickness)	−	−

RA, robust nucleus of the arcopallium.

### Doublecortin Quantification

To quantify the number of newly generated neurons migrating and being recruited to HVC, DCX+ fusiform and spherical cells, as well as the coverage of DCX+ somata and neurites in HVC were quantified. These measurements were not made in RA or Area X. RA has been reported to not actively recruit neurons in adulthood and was devoid of DCX reactivity in all tissue examined. Also, the rate of neuronal recruitment to Area X is not seasonally regulated and falls outside the scope of this study [29].

Images of HVC cross-sections were taken using a 40× objective lens using the microscope set-up mentioned above on 5 adjacent tissue sections (intersection interval = 160 um) in one randomly selected hemisphere as no previous work has reported lateralization in DCX-reactivity in HVC [27], [28]. Pictures were also taken in the ventral nidopallium adjacent to HVC in the same 5 sections. Each picture was compiled as a z-stack using Leica Application Suite from a series of images taken at a regular interval (0.63 µm) throughout the focal depth of the section using a Leica 420D camera. Compiling these photos created an image in which all cells and neurites were in focus.

Images were corrected for brightness/contrast and converted to 32-bit grayscale images using ImageJ software. Fusiform, migratory cells and spherical, post-migratory cells were counted separately. Images were thresholded to highlight immunoreactive somata and neurites from background reactivity. Threshold values were set by an experimenter blind to bird treatments and were confirmed by eye to exclude background reactivity. Next, the *analyze particles* function of ImageJ was used to calculate the percentage of cover of the field of view comprised by immunoreactive somata and neurites.

### Statistical Analyses

All statistical analysis was completed using PASW (SPSS) version 18. To confirm DHT administration, beak color change, syrinx mass, and body mass at sacrifice were compared across treatments, as all three of these traits are androgen-sensitive [30]–[32]. Body mass at sacrifice was also analyzed to confirm E2 administration as E2 treatment is associated with hyperphagia leading to body weight increase [33]. Beak color change was compared using a repeated-measures GLM with a within-subjects factor of time at two levels (d0-d10 or d10-d21) and the between-subjects factors of sex at two levels (M or F), androgen treatment at two levels (DHT or FLUT), and estrogen treatment at two levels (E2 or FAD). Perfused syrinx mass and body mass at sacrifice were analyzed using GLMs in which fixed factors included sex at two levels (M or F), androgen treatment at two levels (DHT or FLUT), and estrogen treatment at two levels (E2 and FAD). Since pre-treatment body mass may influence syrinx mass, these two measures were compared. If a significant correlation was found, analysis of syrinx masses included pre-surgery body mass as a covariate.

An outlier analysis identified birds that were consistently beyond ±2 S.D. of treatment group means on a majority of neuroanatomical traits. Body mass was compared to volume measures in the brain and when significant correlations were found, statistics on these volumes were re-analyzed using pre-surgery weight as a covariate. To investigate potential lateralization, song nuclei volumes were compared across hemispheres using a repeated-measures GLM. The within-subjects factor was hemisphere at two levels (Right or Left) and the between-subjects factors in this analysis were sex at two levels (M or F) and treatment group (at 5 levels). Lateralization was identified in this analysis as an effect of Hemisphere or any interaction including the Hemisphere factor. As this analysis yielded no lateralization effects, all further analysis used the sum of left and right hemisphere neuroanatomical measures.

Based on hormone administration and antagonism, birds were classified by sex on two levels (M or F), androgen treatment on two levels (DHT or FLUT), and estrogen treatment on two levels (E2 or FAD). Subsequent GLM analyses compared groups across these sex, androgen treatment, and estrogen treatment factors. By classifying birds by sex and hormone agonism/antagonism instead of treatment group, comparisons across each factor collapsed across other treatments and greatly increased sample sizes and statistical power. The telencephalon was analyzed for differences in total volume. HVC was analyzed for differences in total volume, volume as a percentage of telencephalon volume, NeuN+ cell count, NeuN+ cell density, DCX+ spherical cells sampled, DCX+ fusiform cells sampled, and DCX+ percent cover. All DCX measure analyses included the corresponding measure in the adjacent nidopallium as a covariate. RA was analyzed for differences in total volume, volume as a percentage of total telencephalon volume, NeuN+ cell count, NeuN+ cell density, and average NeuN+ soma size. Area X was analyzed for differences in total volume and volume as a percentage of total telencephalon volume.

To evaluate the efficacy of a gonadectomy to inhibit T metabolite signaling that may have influenced song-control system growth, all brain measures were compared between the Control and DHT- E2- male treatment group using independent sample t-tests.

## Results

### Outliers

This analysis led to the exclusion of a single bird (female, DHT+ E2+ treatment) detected as an outlier on 5 of 8 anatomical measures. All further analyses excluded data from this animal.

### Treatment Confirmation

#### DHT

At the beginning of the study, beaks were on average 36% (n = 70) yellow prior to surgery. Changes in beak colour (% beak that turned yellow between d0 to d10 and d10 to d21 post-surgery) were compared using a repeated-measures GLM that revealed a significant time x DHT interaction (F_(1,54)_ = 5.4, p<0.05). Post-hoc tests revealed that a greater percentage of the beak turned yellow in birds treated with DHT (mean ± sem = 9.2±2.2%) than birds treated with FLUT (2.3±1.8%) between d10 and d21 (t_31_ = 1.3, p>0.05). Thus, birds treated with androgen continued yellowing their beak over the study, whereas birds treated with FLUT arrested beak yellowing. Males had larger syrinxes (111.0±3.0 mg) than females (78.3±1.6 mg; F_(1,54)_ = 68.2, p<0.01) and birds treated with DHT had larger syrinxes (98.6±3.7×10^−2^ g) than birds treated with FLUT (91.3±3.8×10^−2^ g). Birds treated with DHT gained significantly more mass during treatment (5.6±1.1 g) than birds treated with FLUT (3.0±1.0 g; F_(1,54)_ = 4.2, p<0.05). Last, birds treated with DHT had significantly greater plasma levels of DHT (1.51±0.37 ng/mL) than birds treated with FLUT or control birds (0.18±0.24 ng/mL and 0.21±0.14 ng/mL, respectively; F_(2,62)_ = 154.6, p<0.05).

#### E2

Birds treated with E2 gained significantly more mass during treatment (6.98±0.93 g) than birds treated with FAD (1.64±0.97 g; F_(1,54)_ = 17.2, p<0.01). In addition, birds treated with E2 had significantly greater plasma levels of E2 (1.11±1.21 ng/mL) than birds treated with FAD or control birds (0.01±0.02 ng/mL and 0.04±0.04 ng/mL, respectively; F_(2,62)_ = 15.3, p<0.05).

### Body Mass-brain Relationship

Pre-surgery body mass was significantly correlated with telencephalon volume (r = 0.41, n = 68, p<0.01), HVC volume (as % of telencephalon; r = 0.27, n = 68, p<0.05), Area × volume (r = 0.32, n = 69, p<0.01), and Area × volume as a percentage of telencephalon volume (r = 0.32, n = 68, p<0.05). Analyses on these neuroanatomical measures reported below were repeated using pre-surgery body mass as a covariate. However, inclusion of body mass as a covariate did not alter any of the results, we thus report statistics without the covariate.

### Lateralization

There were no significant main effects of hemisphere for the volumes of the telencephalon or the song-control regions (all p>0.05). Thus, all volumetric analyses below used the sum of left and right hemispheric volumes.

### Neuroanatomy

#### Telencephalon

Males had significantly larger telencephalons (9.09±0.10×10^2^ mm^3^) than females (8.33±0.14×10^2^ mm^3^; F_(1,53)_ = 19.3, p<0.01). There were no significant effects of E2, DHT, or interactions (all p>0.05).

#### HVC

Males had significantly larger HVC volumes than females (F_(1,53)_ = 79.7, p<0.01; Figure 1B). However, there were no significant DHT, E2, or interaction effects (all p>0.05) on HVC volume. When HVC volumes were reanalyzed as a percentage of telencephalon volume, similar results were found (data not shown).

Males had significantly more NeuN+ cells in HVC than females (F_(1,53)_ = 42.7, p<0.01; Figure 1C). Although not significant, this analysis also yielded a notable trend in which birds treated with E2 tended to have more NeuN+ cells in HVC than birds treated with FAD (F_(1,53)_ = 3.2, p = 0.081; Figure 1C). Last, there were no significant DHT or interaction effects (all p>0.05).

Females had a greater NeuN+ cell density in HVC than males (F_(1,53)_ = 19.9, p<0.01; Figure 1D). Additionally, birds treated with E2 had significantly lower NeuN+ cell densities in HVC than birds treated with FAD (F_(1,53)_ = 5.0, p<0.05; Figure 1D). No significant DHT or interaction effect was found in this analysis (p>0.05).

#### RA

Males had significantly larger RA volumes than females (F_(1,53)_ = 79.7, p<0.01; Figure 2B). Additionally, there was a significant sex x DHT x E2 interaction (F_(1,53)_ = 4.7, p<0.05). Post-hoc analyses indicated a DHT x E2 interaction found only in males (F_(1,27)_ = 3.9, p = 0.058) as being responsible for the original interaction. Further analysis of this interaction using t-tests suggests that this trend is likely due to male birds treated with FLUT and FAD having smaller RA volumes (1.26±0.14 mm^3^) than males treated with DHT and FAD (1.72±0.15 mm^3^). There were no significant main effects of E2, DHT, or other interaction effects (all p>0.05). When RA volumes were reanalyzed as a percentage of telencephalon volume, similar results were found (data not shown). Thus, there was limited evidence that DHT maintained RA size, but only in males treated with FAD.

Males had more NeuN+ cells in RA than females (F_(1,53)_ = 54.0, p<0.01; Figure 2C). Birds treated with FLUT had fewer NeuN+ cells in RA (1.83±0.07×10^4^ cells) than birds treated with DHT (2.05±0.09×10^4^ cells; F_(1,53)_ = 4.5, p<0.05). However, there was also a significant sex x DHT interaction, so we compared the effects of DHT separately for each sex. Males treated with FLUT had fewer NeuN+ cells in RA than males treated with DHT (t_29_ = 2.8, p = 0.01; Figure 2C), but females were not found to differ significantly in RA NeuN+ cell count based on androgen treatment (p>0.05). Thus the main effect of DHT on RA NeuN+ cell number was driven primarily by an effect in males.

In addition to the effects above, there was a significant DHT × E2 interaction for NeuN+ cell number in RA. Post-hoc analysis of this interaction indicated that males treated with FLUT and FAD had significantly fewer NeuN+ cells in RA when compared to birds treated with DHT and FAD (t_30_ = 2.2, p<0.05; Figure 2C). Conversely, no significant effect of DHT treatment was found in birds treated with E2 (p>0.05). Last, there were no significant E2 or other interaction effects (all p>0.05). Thus in addition to the main effect of DHT on NeuN+ cell number in RA being observed primarily in males, this effect was also primarily observed in birds not treated with E2. Females had a greater NeuN+ cell density in RA than males (F_(1,53)_ = 53.1, p<0.01; Figure 2D). No significant DHT, E2 or interaction effect was found on NeuN+ cell density in RA (p>0.05).

A non-significant trend was found in which birds treated with E2 had larger average soma size in RA (0.118±0.007×10^−3^ mm^2^) than birds treated with FAD (0.113±0.006×10^−3^ mm^2^; F_(1,53)_ = 3.4, p = 0.069). Analysis of average RA soma size also yielded a significant DHT x E2 interaction (F_(1,53)_ = 3.4, p<0.05). Follow-up analysis demonstrated that, in birds treated with FLUT, concurrent treatment with FAD reduced average RA soma size significantly when compared to birds treated with E2 (F_(1,29)_ = 5.6, p<0.05; Figure 2E). Sex, DHT, nor any other interaction of these factors significantly affected RA soma size (all p>0.05).

#### Area X

Males had significantly larger Area X volumes than females (F_(1,53)_ = 190.6, p<0.01; Figure 3B). Additionally, a significant sex x DHT × E2 interaction was found (F_(1,53)_ = 4.3, p<0.05). Post-hoc analysis revealed a significant DHT × E2 interaction in female birds only (F_(1,53)_ = 5.8, p<0.05; in males, p>0.05). Independent sample t-tests testing for all possible pairwise comparisons resulted in no significant effects of DHT, E2, or an interaction of these two effects (p>0.05). This lack of effects suggests the original sex × DHT × E2 interaction effect was due to strong, but opposing trends in female treatment groups.

### Neurogenesis

#### DCX+ fusiform cells

**Figure 4:**
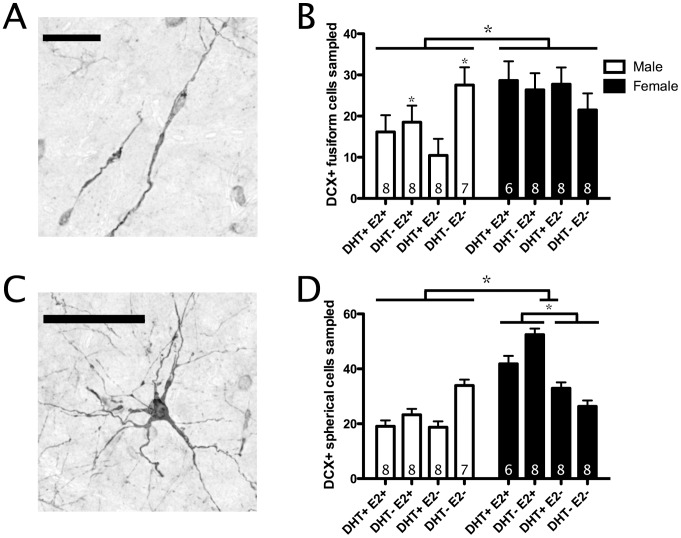
Effects of testosterone metabolites on doublecortin-stained (DCX+) cell recruitment to HVC. A) Photomicrograph of DCX+ fusiform cell. Scale bar = 50 µm. B) Number of DCX+ fusiform cells sampled in HVC across treatments. C) Photomicrograph of DCX+ spherical cell. Scale bar = 50 µm. B) Number of DCX+ spherical cells sampled in HVC across treatments. In all figures, bars represent estimated marginal mean ± SEM using the same measurement in the adjacent nidopallium as a covariate. * <0.05. In B), asterisks without connecting lines mark significant differences found using post-hoc analysis.

Females recruited more DCX+ fusiform cells into HVC than males (F_(1,52)_ = 7.4, p<0.01; Figure 4B). Furthermore, a significant sex × DHT interaction was found (F_(1,52)_ = 5.6, p<0.05). Post-hoc analysis indicated a significant effect of DHT only in males (in females, p>0.05). Specifically, males treated with DHT had fewer DCX+ fusiform cells in HVC than male birds treated with FLUT (F_(1,26)_ = 4.8, p<0.05; Figure 4B). There were no significant E2 or interaction effects on DCX+ fusiform cell count in HVC (p>0.05).

#### DCX+ spherical cells

Females recruited more DCX+ spherical cells into HVC than males (F_(1,52)_ = 10.6, p<0.01; Figure 4D). Furthermore, a significant sex x E2 interaction was found. Post-hoc analysis indicated a significant effect of E2 in females only (in males, p>0.05). In females, birds treated with E2 recruited more DCX+ spherical cells into HVC than female birds treated with FAD (F_(1,25)_ = 7.1, p<0.05; Figure 4D). In males, E2 treatment had no significant effect on DCX+ spherical cell number in HVC (p>0.05). There were no significant DHT or interaction effects on DCX+ spherical cell number in HVC (p>0.05).

Finally, analysis of DCX immunoreactivity measured by percent cover of the field of view yielded no significant effects of Sex, DHT, E2, or any interaction of these factors (all p>0.05).

### Effects of Gonadectomy

**Figure 5:**
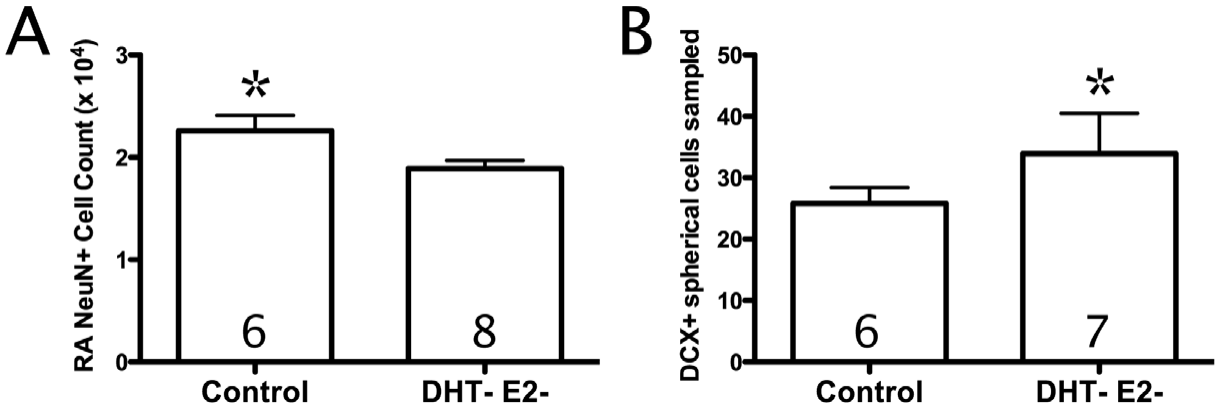
Neuroplastic differences between castrated male starlings and DHT- E2- male starlings. A) NeuN-stained cell count in. Bars represent mean ± SEM. B) DCX+ spherical cells sampled in HVC. Bars represent estimated marginal mean ± SEM using DCX+ spherical cells sampled in the adjacent nidopallium as a covariate. * <0.05. DCX+, doublecortin-stained.

To determine the effects of a gonadectomy relative to gonadectomy and hormone antagonism we compared the control group (n = 6) to the male birds in the FLUT+FAD (n = 8) group using a GLM for all neuroanatomical measures included in this study. Treatment with FLUT and FAD significantly reduced NeuN+ cell count in RA when compared with the Control group (F_(1,12)_ = 5.4, p<0.05; Figure 5A) and significantly increased the number of DCX+ spherical cells in HVC compared with the Control group F_(1,10)_ = 5.2, p<0.05; Figure 5B). However, there were no significant differences between these two groups on the other measures (all p>0.05).

## Discussion

**Figure 6:**
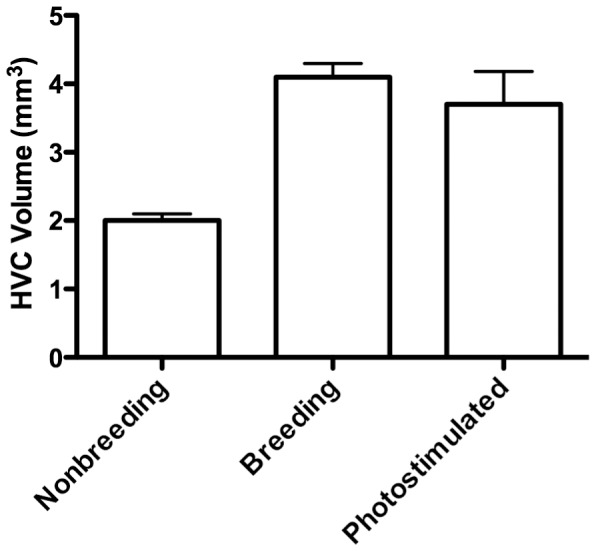
HVC volumes in male free-living European starlings compared to male HVC volume in this study. Bars represent mean ± SEM. Data for Belgian European starlings in nonbreeding (September) and breeding (April) conditions from [56]. Data for photostimulated birds from mean male HVC volume in this study regardless of hormonal manipulation.

We used pharmacological manipulations to isolate androgenic and estrogenic contributions to song-control system growth during photostimulation in adult European starlings. However, neither the isolated nor simultaneous activation or inhibition of androgen and estrogen signaling pathways were responsible for the induction of song-control system plasticity previously observed following photostimulation during the breeding season and treatment with T. Comparison with previous studies (Figure 6) indicates that all of our treatment groups had large HVC volumes characteristic of breeding condition. Thus, it appears photostimulation was able to increase HVC volume independently of our hormonal manipulations; however, estrogen treatments did have additional effects inducing HVC growth.

### Testosterone vs. Photoperiod effects on HVC

T has long been implicated as the primary physiological cue that induces HVC growth in temperate-zone songbirds photostimulated in the breeding season. Both seasonal and experimental manipulations have demonstrated T’s consistent role in inducing HVC growth, primarily through enhanced neuronal recruitment. Furthermore, the importance of both androgenic and estrogenic signaling pathways has been investigated: a recent study in photosensitive adult female canaries demonstrated that HVC growth stimulated by T treatment was only possible through the synergy of DHT and E2 signaling [16]. Alternatively, in photostimulated adult male white-crowned sparrows, treatment with DHT, E2, DHT+E2, or T would induce maximal HVC volume growth, suggesting redundant effects [9]. In our study, we found that photostimulation-induced HVC growth was not affected by treatment with, or antagonism of DHT or E2 in adult male and female European starlings. When comparing the HVC volumes of all of our treatment groups to the volumes of HVC across seasons in free-living starlings (Figure 6), we found that all of our treatment groups exhibited enlarged HVCs characteristic of birds in breeding condition. Bernard and Ball [7] found that the photoperiodic state of starlings can significantly modulate the efficacy of T treatment in inducing HVC growth. Specifically, photorefractoriness completely inhibited the ability for T to significantly increase HVC volume. In our study, adult starlings were photostimulated and the DHT and E2 signaling stimulated by exogenous T treatment had no influence on the enlarged HVC volumes found in all treatment groups. Similarly, male American tree sparrows (*Spizella arborea*) exhibited HVC growth during photostimulation even following T-signaling reduction via castration [34].

Our finding that HVC and RA grew in the absence of T metabolite signaling is surprising given the extensive work emphasizing the central role of T signaling in seasonal song nuclei growth [1]. Without being able to sample hormone levels locally in the song-control system, we cannot entirely rule out the possibility that our pharmacological treatments did not entirely block physiologically-significant levels of DHT and E2 signaling. A major problem with the use of pharmacological antagonists in birds chronically is the stimulation of feedback signaling and upregulation of hormone production in response to chronically low hormone levels. For example, Fusani et al. [35] demonstrate that chronic FLUT administration reduces courtship behaviour in male golden-collared manakins at first, but that this effect disappears and even reverses after 3 weeks of treatment. We anticipated that compensatory production of gonadal hormones would be avoided in our study by gonadectomizing all of our birds. Despite this, the possibility remains that neurosteroid production of DHT and E2 may upregulate to overcome chronic signaling inhibition by FLUT and FAD, respectively. Although all of our systemic measures of hormone administration confirmed our treatments, future studies should specifically investigate local levels of hormones being manipulated in the brain structures of interest to confirm treatment.

Our interpretation also excludes the possibility that non-steroidal signals may act to enhance song nuclei volume independent from DHT and E2 signaling. Melatonin receptor density in Area X is known to fluctuate with reproductive condition independently of photoperiodic state, potentially used to entrain song-control system neuroplasticity to seasonal changes in photoperiod [36]. Specifically, photostimulated birds exposed to long days were found to have a lower density of melatonin receptors than photosensitive or photorefractory birds, creating a dissociation between cytoarchitecture and day length. In our study, however, our surprising results were found in HVC, which is known to have downstream stimulatory effects on Area X and RA and not the other way around [37], [38]. Additionally, we confirmed standardized reproductive states in our birds by visual confirmation of regressed gonads during surgery and standard photostimulation treatment for all birds.

Our findings suggest the importance of T signaling in inducing song-control system growth in the starling may be seasonally regulated. Furthermore, our findings also suggest that the seasonal regulation of T-induced song-control system growth differs between species, as DHT and E2 were capable of inducing HVC volume growth in photostimulated sparrows (above).

### Effects of Androgens

We predicted that androgen receptor (ant)agonism would influence song-control system growth by increasing the recruitment of neurons to HVC. During the breeding season HVC volume increase is predominantly characterized by an increase in neuron number, and increased neuronal recruitment to HVC using T treatment has been demonstrated in multiple songbird species [1]. In our study, enhanced neuronal recruitment would be observed as an increase in DCX+ cells in HVC. No such increase was observed with DHT treatment. Surprisingly, DHT reduced migratory DCX+ neuron count in the HVC of males (Figure 4B). This reduction could be due to DHT i) impairing neuroproliferation and/or migration of neurons to HVC, ii) reducing the time recruited neurons express DCX, or iii) increasing the turnover rate of new neurons in HVC. Recent studies suggest the last of these three possibilities as most likely, since i) neuronal migration is non-specific to HVC in comparison with surrounding nidopallium [39] and no suppressive effect of DHT on nidopallium DCX+ cell sampling was found (data not shown) and ii) an increased number of NeuN+ neurons in HVC indicative of accelerated maturation was not observed. Our study is the first to report a negative effect of androgen treatment on new neuron migration to HVC. It should be noted that Absil et al. [24] reported increased ventricular neuroproliferation with T treatment without a coincident increase in HVC neuronal recruitment in European starlings. We did not investigate ventricular neuroproliferation as this may not reflect levels of neuronal incorporation in HVC. Thus, future work is needed to determine the effects of DHT treatment on ventricular proliferation.

### Effects of Estrogens

We predicted that estrogen receptor (ant)agonism would influence HVC by reducing neuron turnover to increase HVC neuron recruitment and, ultimately, cell count. Rasika et al. [40] reported the neuroprotective effects of T treatment on survival of post-mitotic cells in HVC. Furthermore, Hidalgo et al. [17] demonstrated that most of these effects of T were replicated by E2 treatment, likely mediated through the expression of brain-derived neurotrophic factor [3], [41]. If E2 reduced turnover rates of mature HVC neurons, we predicted that E2 treatment would increase NeuN+ cell count in HVC. Alternatively, if E2 reduced turnover in newly recruited neurons, we expected E2 treatment to increase maturing DCX+ cell count in HVC. Our results were consistent with the second of these two mechanisms, but only in female starlings: E2 treatment increased the number of spherical DCX+ cells sampled the female HVC (Figure 4D). This finding supports the prediction that T treatment enhances new neuron survival in HVC via its aromatization into E2. Additional evidence of E2’s role in reducing mature cell turnover was found in a non-significant trend for E2 treatment to increase HVC NeuN+ cell count. This trend may have reached significance given a longer survival time, as 21 days is likely an insufficient period for many neurons recruited to HVC during treatment to begin terminal differentiation and begin expressing NeuN [25]. Our findings corroborate the robust ability of E2 treatment to enhance survival of neurons in HVC.

Additionally, E2 treatment significantly decreased NeuN+ cell density in HVC (Figure 1D). This finding indirectly supports previous reports of E2’s ability to enhance HVC vascularization and subsequently reduce neuronal density [3], [17].

It is important to address that, although E2 treatment enhanced neuronal recruitment and reduced NeuN+ cell density in HVC, the overall volume of HVC was not found to significantly increase with E2 treatment. This discrepancy in cytoarchitecture and volumetric measurement may have been due to the insensitivity of gross volumetric measures to sense smaller changes in neuroanatomy or the myriad of other neuroplastic characterstics in HVC that we did not measure (e.g., cell turnover and glial density) which may have compensated for changes in neuronal recruitment and NeuN+ cell density to maintain HVC volume. The former of these two possibilities seems most likely as a non-significant trend for HVC volume increase was noted in female starlings treated with E2 (the only birds to experience both the decrease in NeuN+ cell density and enhanced neuron recruitment in HVC).

### Sex Differences

Prior studies report similar, but attenuated seasonal changes in female HVC size when compared to males [42], [43]. Therefore, we predicted females would exhibit similar, but attenuated neuroplasticity to T metabolite treatment compared to males. In contrast, we found multiple sex-specific effects. In males only, DHT treatment decreased the number of migratory DCX+ neurons in HVC (Figure 4B). In females only, E2 treatment increased the number of maturing DCX+ neurons in HVC (Figure 4D). Interestingly, females had significantly more DCX+ cells in HVC than males (Figure 4B, D). Alternatively, prior studies report a male-bias in neuronal recruitment to HVC in zebra finches [44], [45] and female canaries (not to significance; [28]). Our study is the first report of a species exhibiting greater neuronal recruitment in female HVC. As female starlings were also found to have fewer neurons in HVC, we can also conclude that female starlings must have a higher turnover rate of neurons in HVC. Current hypotheses of neuronal replacement in HVC posit that newly HVC neurons play a role in song learning [46] or song performance [1], [5]. Although comparative evidence supporting both of these hypotheses exists for males of several songbird species, neither of these hypotheses incorporates potential functional sex differences in neuronal recruitment to HVC and the wide spectrum of female singing behaviors across species [47]. Starling song is open-ended and characterized by male dominance in song production and complexity year round [48]. If HVC neuron recruitment functionally contributes to song, it would be interesting for future studies to examine the modification of adult birdsong across both species and sex in comparison with measures of neurogenesis and neuronal recruitment in the song-control system. Alternatively, this significant female bias in neuronal recruitment in HVC may be related to song perception instead of production. This is particularly of note as female starlings have been shown capable of recognizing individual male starlings by multiple, different songs [49].

Males were found to have a larger volume telencephalon, HVC, RA, and Area X than females (Figure 1B, 2B, 3B). Male-biased sex differences in song-control system volumes appear consistently across multiple songbird species [50]. Few studies have investigated the cytoarchitecture underlying these sex differences; however, in marsh wrens, male RA contains more cells at a lower density than females [51]. In this study, we provide some of the first cytoarchitectonic sex differences in the song-control system as both a greater NeuN+ cell count and a lower NeuN+ cell density in male starlings’ HVC and RA compared with females (Figure 1B–C; 2B–C).

### Efficacy of Gonadectomy

Previous studies investigating T-induced neuroplasticity often used gonadectomized birds as controls. However, nongonadal sources of steroids continue to produce T following gonadectomy [52], [53]. By comparing castrates to our DHT- E2- group we found that nongonadal androgens maintained RA NeuN+ cell count and syrinx mass and nongonadal androgens and estrogens maintained RA volume (strong trend) and soma size. Since RA has never been reported to recruit new neurons in adulthood, this means that any neurons lost in the absence of androgen signaling would never be replaced in these starlings. These findings suggest an active role for a basal level of nongonadal T in maintenance of the RA nxIIts syrinx pathway involved in motor production of song. Whereas the seasonal growth and regression of RA has been shown to be controlled by the trans-synaptic transmission of neurotrophins from HVC [37], [38], our study is the first to provide evidence of a role for neuroandrogens to maintain the structurally integrity of RA independently of HVC. We do not believe such a loss of cells in RA would occur in a wild bird along with seasonal and hormonal changes in physiology. Instead, we interpret this reduction in RA cell number as the cytoarchitectonic consequence of removing basal, nongonadal T metabolites that could only be accomplished using pharmacological manipulation. Interestingly, Grisham et al. [54] demonstrated that chronic treatment with FLUT in developing male zebra finches reduced the number of neurons in RA as well, suggesting a similar mechanism maintaining and/or determining RA neuron number may be shared between development and adulthood.

Additionally, gonadectomy and antagonist administration led to greater numbers of maturing DCX+ cells recruited to HVC than gonadectomy alone. In conjunction with the observed DHT suppression of neuronal recruitment, this finding further corroborates the possibility that androgenic metabolites during photostimulation may suppress neuronal recruitment to HVC. These findings indicate caution must be taken when selecting control groups in studies of neuroplasticity. Since many prior studies attempting to isolate the influences of androgen and estrogen on song-control system neuroplasticity did not use antagonists in control treatments, the potential interaction with nongonadal steroids cannot be ruled out. For example, the effects of DHT on HVC size may depend on low levels of nongonadal estrogens.

### Conclusions

The song-control system exhibits extreme seasonal anatomical changes, and has become a model system for neuroplasticity [1]. Gonadal testosterone is clearly indicated as an important modulator of this plasticity. Our study is one of the most comprehensive to date in terms of sample size, inclusion of both sexes and range of anatomical and cellular measures. We found estrogen-mediated effects on HVC plasticity, but no isolated effects of androgens. We also found that T signaling pathways may play an attenuated role during photostimulation in starlings in comparison with other photoperiodic states and songbird species. Moreover, we found sex-specific neuroplastic responses to androgens and estrogens. Understanding the complex suite of cellular and anatomical changes in the brain and how they are regulated by androgens and estrogens, as well as other hormones and neuromodulators, will require both further studies that isolate particular signaling pathways as well as a consideration of species and sex differences in these mechanisms.
